# Investigation on the Ageing Behaviour of High-Modulus Modified Bitumen Based on Rheological and Chemical Approaches

**DOI:** 10.3390/ma18061332

**Published:** 2025-03-18

**Authors:** Xuemao Feng, Xin Li, Mingchen Li, Liping Liu, Zhenbang Cheng, Zhanchuang Han

**Affiliations:** 1 School of Civil Engineering, Central South University, Changsha 410083, China; xmfeng@csu.edu.cn; 2 Guangxi New Development Transportation Group Co., Nanning 530029, China; knightkt@126.com; 3 School of Infrastructure Engineering, Dalian University of Technology, Dalian 116024, China; 4 The Key Laboratory of Road and Traffic Engineering, Ministry of Education, Tongji University, Shanghai 201804, China; 2331699@tongji.edu.cn; 5 Department of Civil and Environment Engineering, National University of Singapore, Singapore 117576, Singapore; ceev320@nus.edu.sg; 6 China First Highway Engineering Group Co., Ltd., Beijing 100024, China

**Keywords:** HMB, ageing temperature, ageing behaviour, molecular weight distribution, rheological properties

## Abstract

With outstanding resistance for permanent deformation, high-modulus modified bitumen (HMB) has garnered widespread attention in recent years and has been employed in the construction of bitumen pavements across various regions. However, limited research exists on the ageing behaviour of HMB, and conventional short-term ageing protocols for bitumen may not be applicable to HMB due to its exceptionally high viscosity. Therefore, this study aims to assess the ageing behaviour of HMB and propose a suitable short-term ageing process for HMB utilizing dynamic shear rheometer (DSR) and gel permeation chromatography (GPC) approaches. For comparison purposes, the ageing behaviour of a type of SBS-modified bitumen and a kind of base bitumen were also analyzed. Initially, the study involved a comparison of the properties of bitumen subjected to short-term ageing at various temperatures and those of bitumen within mixtures undergoing short-term oven ageing tests. Subsequently, both the chemical and rheological properties of bitumen under diverse ageing conditions were examined. Finally, investigations were conducted to establish relationships between rheological properties and the molecular weight distribution of HMB. The reported results indicate that the suggested ageing temperature for the thin-film oven test (TFOT) should be increased to 193 °C for HMB, achieving a more accurate simulation of short-term ageing in HMB mixtures during on-site mixing, transport, and paving processes. Compared to base bitumen and SBS-modified bitumen, HMB exhibits superior ageing resistance. Furthermore, the molecular weight distribution of HMB is strongly correlated with its rheological properties. This correlation offers a promising approach to predict the rheological properties of bitumen in HMB mixtures by directly analyzing the chemical molecular weight distribution of the binders, thereby eliminating the need for an extraction process.

## 1. Introduction

Due to advantages such as a short construction period, ease of maintenance and repair, and enhanced driving comfort, the utilization of bitumen pavements in pavement engineering is increasingly gaining popularity in China [1]. During the service process, bitumen pavements are vulnerable to damage such as rutting damage under the integrated action of the external environment and traffic loads [2,3,4,5]. High-modulus bitumen (HMB) mixtures are recognized as potent paving materials to address the growing concerns of rutting [6,7,8,9,10]. The concept of HMB mixtures was first introduced in France in the 1980s, which stipulated that the dynamic modulus E* (15 °C, 10 Hz) of HMB mixtures should exceed 14,000 MPa [6]. Due to the high dynamic modulus of the bitumen mixture, it can effectively reduce the occurrence of plastic deformation of the pavement, thus effectively inhibiting the occurrence of rutting damage. For their excellent stiffness modulus and resistance to deformation, HMB mixtures have been progressively adopted in the construction of bitumen pavements [11,12,13,14,15]. Currently, 50% of bitumen pavements in France apply HMB mixtures. The application of HMB mixtures provides high structural strength to bitumen pavements, increasing the service life and reducing the thickness of bitumen pavements.

The production of HMB mixtures places high demands on the quality of the bitumen. Currently, low-grade bitumen with low penetration is commonly used as a binder in HMB mixtures [16,17]. However, the use of low-penetration base bitumen compromises the moisture susceptibility and low-temperature performance of HMB mixtures. As an alternative, the incorporation of pure natural bitumen (PNB) into base bitumen to produce HMB is currently used in the production of HMB mixtures in many regions [18,19,20]. Compared with low-grade base bitumen, HMB prepared by PNB has better high-temperature rutting performance and moisture damage resistance. It is possible to enhance the capacity of the bitumen mixture to resist permanent deformation without compromising the low-temperature performance of the bitumen mixture. Thus, HMB has broad development prospects in pavement engineering.

So far, quite a lot of research has been performed on HMB produced with PNB [21,22,23,24,25,26,27,28]. For instance, Wang et al. [21] compared the performance of base bitumen, SBS-modified bitumen, and high-modulus modified mixtures produced with PNB. The reported results showed that among the three types of bitumen mixtures, the PNB-modified bitumen mixture exhibited the best high-temperature performance, water susceptibility, and fatigue performance. However, research focused on the ageing behaviour of HMB is scarce. During the application of bitumen pavements, bitumen in pavements is affected by oxidative ageing in the presence of several environmental causes [29,30,31]. The occurrence of ageing phenomena results in an increase in oxygen-containing functional group contents in the bitumen’s molecular structure and a decrease in the light fraction. In addition, after ageing, bitumen will turn stiff and be prone to cracking under vehicle loads at low temperatures, which seriously affects the service life of bitumen pavements [32,33]. Although the modification of base bitumen using PNB improves the performance of bitumen, it still fails to avoid the problem of ageing. It is of great importance to investigate the ageing characteristics of HMB.

For this purpose, the thin-film oven test (TFOT) is usually performed in China to simulate the short-term ageing process of bitumen. Additionally, the pressure ageing vessel (PAV) test is subsequently carried out to simulate the long-term ageing process of bitumen binders during the pavement service life. For the TFOT procedure, the bitumen in the container is aged in a 163 °C oven for 5 h. However, the ageing temperature of 163 °C is feasible for base bitumen but is somewhat low for polymer-modified bitumen, which has a higher viscosity [34,35]. According to the survey results of the NCHRP project, the short-term ageing procedure of the modified bitumen was one of the most problematic issues [36,37]. Xing et al. [38] compared the rheological properties of extracted bitumen in short-term ageing bitumen mixtures with that of high-viscosity modified bitumen aged by the TFOT under different short-term ageing temperatures, and proposed that for high-viscosity modified bitumen, 178 °C was the suitable ageing temperature in the TFOT. Similarly, Yan et al. [35] concluded that the suitable short-term ageing temperature for polymer-modified bitumen with 4.5% SBS was 178 °C. To find TFOT ageing temperatures suitable for HMB, the most direct method is to compare the properties of recovered bitumen binders in short-term-aged bitumen mixtures with those of bitumen short-term-aged under different temperatures in the TFOT. Unfortunately, the method of extraction and recovery is not applicable for HMB prepared from PNB since the ash in PNB cannot be extracted and recovered. This calls for a method to test the properties of the binder in HMB mixtures directly without the need for extraction and recovery processes. In recent years, the application of some advanced observation techniques in the field of bitumen materials has provided the possibility to solve the above problem [39,40,41]. Among these advanced observation techniques, gel permeation chromatography (GPC) is a method to test the molecular weight and molecular size distribution of polymer materials, which is now widely used in the study of modification mechanisms, ageing, and recycling of bitumen materials [42,43]. Using GPC, Li et al. [44] introduced a novel method to directly measure the properties of the binder in the mixtures, eliminating the processes of extraction and recovery. The method proposed in this study provides assistance in directly measuring the properties of HMB in short-term-aged mixtures.

Given this, this study aims to propose an appropriate short-term ageing scheme for HMB and investigate the ageing behaviour of HMB. To achieve the above objectives, HMB was first short-term-aged by the TFOT under different temperatures. And the molecular weight distribution of aged modified bitumen was measured via the GPC test. At the same time, a short-term-aged HMB mixture was produced. Through the comparison of aged modified bitumen’s properties and those of binders in the short-term-aged mixtures, the appropriate ageing temperature in the TFOT for HMB was determined. Following this, the short-term-aged modified bitumen was subsequently subjected to PAV ageing. Finally, the long-term ageing behaviour of HMB was investigated via DSR and GPC tests. For comparison, the ageing behaviour of a type of base bitumen and a type of SBS-modified bitumen was also analyzed.

## 2. Objectives

The main objectives of the present study are as follows:

1. Introduce an appropriate short-term ageing scheme for HMB.
2. Analyze the ageing behaviour of HMB in terms of molecular size and rheological properties.
3. Establish a correlation between the chemical and rheological properties of HMB after prolonged ageing.

## 3. Materials and Methods

### 3.1. Materials

Three types of bitumen were adopted in this study, including a type of base bitumen, a type of SBS-modified bitumen, and a kind of commercial HMB containing 20% natural bitumen. It is noteworthy that the HMB used in this study was prepared from base bitumen with the incorporation of pure natural bitumen. Considering the cost of pure natural bitumen, this method is currently one of the commonly used solutions for producing HMB. The basic properties of these bitumen samples are tested in reference to *Standard Test Methods of Bitumen and Bituminous Mixtures for Highway Engineering* and listed in Table 1.

**Figure 1:**
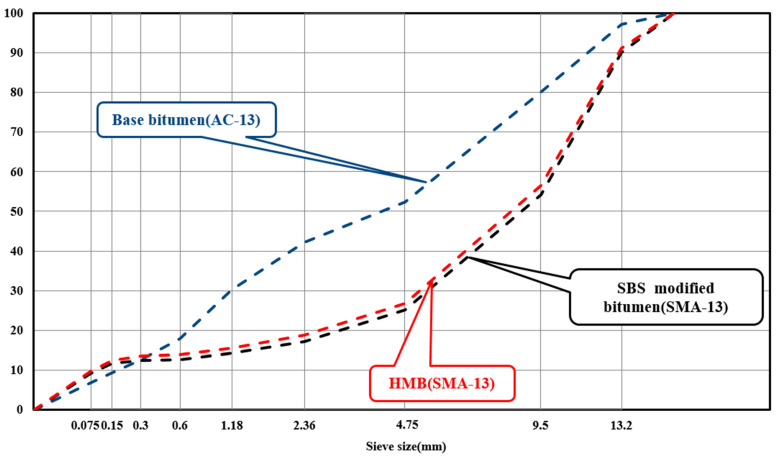
Gradation curves of prepared mixtures.

Limestone (0~3 mm, 3~5 mm) and basalt (5~10 mm, 10~15 mm) were adopted as aggregates in this study. To simulate the practical application, the gradations for three kinds of bitumen were selected as AC-13 (for base bitumen) and SMA-13 (for SBS-modified bitumen and HMB). The gradation curves of these three mixtures are shown in Figure 1. The bitumen mixtures were designed via the Marshall design method. It was calculated that the asphalt content was 4.5% for the base bitumen mixture and 6.5% for the SBS-modified bitumen and HMB mixtures. In addition, to mimic the mixing process of bitumen mixtures on site, the mixing temperatures of base bitumen, SBS-modified bitumen, and HMB mixtures were set to 165 °C, 175 °C, and 185 °C, respectively.

**Figure 2:**
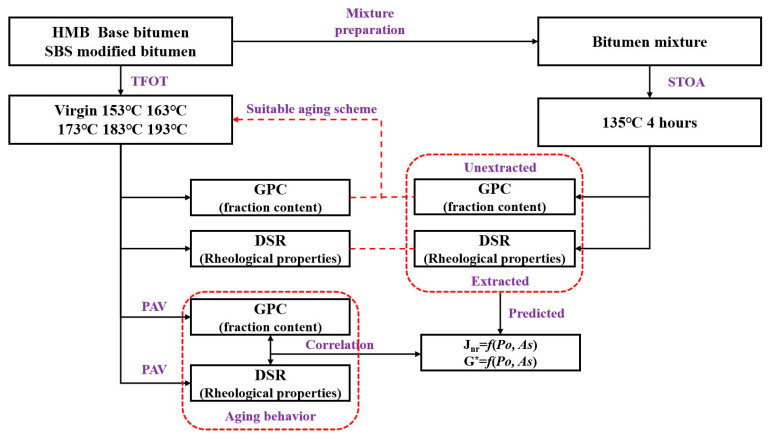
Flowchart of this study.

The flowchart of the present study is shown in Figure 2.

### 3.2. Ageing Scheme

#### 3.2.1. Ageing Scheme for Bitumen

For the ageing protocol of bitumen, TFOT and PAV tests were carried out in this study to prepare the bitumen under various ageing levels. As mentioned above, the regular short-term ageing temperatures do not apply to modified bitumen. To investigate a suitable temperature for HMB, four short-term ageing temperatures were set from 165 °C to 195 °C, with a gradient of 10 °C. For comparison, gradient temperature short-term ageing tests were also set up for base bitumen and SBS-modified bitumen, with ageing temperatures ranging from 155 °C to 185 °C, with a gradient of 10 °C. Once the short-term ageing temperatures were determined, additional 20, 40, and 60 h PAV tests were performed on the bitumen after short-term ageing to obtain the bitumen under different ageing conditions. TFOT and PAV tests were performed with reference to *Standard Test Methods of Bitumen and Bituminous Mixtures for Highway Engineering* (JTG E20-2011) in China [45]. Following ageing tests, residues were taken for property testing. Table 2 lists the ageing protocol and identification of the prepared bitumen.

#### 3.2.2. Ageing Scheme for Bitumen Mixture

For comparison, bitumen mixtures produced with three different bitumen types were subjected to the short-term oven ageing (STOA) test according to AASHTO R 30-02 [46]. In detail, after the mixing of the mixture was complete, the loose mixtures were then placed in the tray. The thickness of the loose mixture in the tray was controlled to about 35 mm. The tray was then placed into a forced draft oven at 135 °C for 4 h. The loose mixture was tipped every half hour to control the uniformity of ageing. After the STOA test, the loose mixture was collected for the GPC test, and the bitumen in the loose mixture was extracted and recovered according to ASTM D1856-21 [47] for DSR tests. This STOA scheme has been confirmed to successfully simulate the short-term ageing behaviour of bitumen mixtures in practical production under different production temperatures, bitumen types, aggregate types, and climatic conditions.

### 3.3. GPC Test

**Figure 3:**
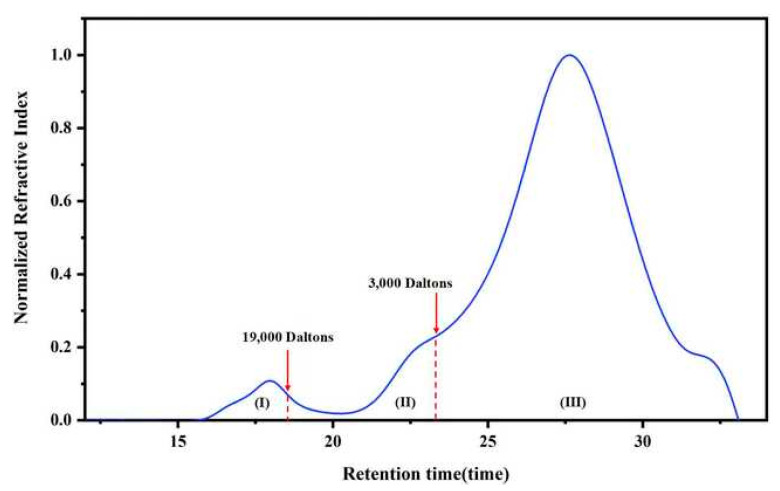
A typical GPC chromatogram of a bitumen sample.

Waters 1515 Isocratic high-performance liquid chromatography was adopted to analyze the molecular weight and molecular weight distribution of the bitumen sample under different ageing conditions. And tetrahydrofuran (THF) was adopted as the mobile phase. During the test, the sample slowly flowed into the column with some porous gel at 1 mL/min under the action of the mobile phase. The smaller the size of the molecules in the sample, the easier it was to spread them into the pores of the gel column, while the larger molecules could pass straight across. As such, small molecules took longer to pass through the gel column than large molecules in the GPC test. A refractive index detector detected the difference in refractive index from the reference flow path towards the sample, and the included computer software recorded the retention time and refractive index data of the measured sample. Figure 3 presents a typical chromatogram of a representative bitumen in the GPC test. By establishing the calibration curve, the sample’s retention time was transformed into the molecular weight of the sample.

#### 3.3.1. Sample Preparation Approach for GPC Test

To prepare GPC samples of bitumen and bitumen mixtures under different ageing conditions, bitumen and loose bitumen mixtures were first weighed using an analytical balance. Then, the weighed sample was dissolved in 10 mL of THF, and the concentration of bitumen in THF was controlled at 3.5 g/L. After complete dissolution of the bitumen, the solution (bitumen + THF) was filtered via a 0.45 μm filter. Finally, the filtered solution was collected for testing.

#### 3.3.2. GPC Chromatogram Quantification Method

With reference to existing studies, the GPC chromatogram of different samples was quantitatively analyzed [48]. Specifically, with a cut-off point of 3000 and 19,000 Daltons, the GPC chromatograms were grouped into polymer (*Po*%), asphaltene (*As*%), and maltene (*Ma*%) fractions according to the range of molecular weights. The content of each fraction in the sample can be calculated via the following equation. Three parallel tests were conducted for each set of samples.

(1) $$Po(\%)=\frac{Area_{p}}{Area_{T}}\times 100\%$$

(2) $$As(\%)=\frac{Area_{A}}{Area_{T}}\times 100\%$$

(3) $$Ma(\%)=\frac{Area_{M}}{Area_{T}}\times 100\%$$

where *Area_P_* is the area of the polymer region in the chromatogram; *Area_A_* is the area of the asphaltene region in the chromatogram; *Area_M_* is the area of the maltene region in the chromatogram; and *Area_T_* is the total area of the chromatogram.

### 3.4. Temperature Sweep (TS) Test

The TS test was performed in DSR at 58 °C, 64 °C, 70 °C, and 76 °C to measure the complex modulus (G*) and phase angle (δ) of the prepared bitumen samples. During the TS test, a sinusoidal oscillatory load with a frequency of 10 rad/s was applied in a controlled strain mode, and the strain levels were all set to 10%. Three parallel tests were conducted for each set of samples.

### 3.5. Multiple Stress Creep and Recovery (MSCR) Test

The MSCR test was performed according to ASSHTO TP-350 [49] on the prepared bitumen samples at 58 °C, 64 °C, 70 °C, and 76 °C. During the test, 10 creep–recovery cycles were loaded on the samples at 3.2 kPa. Each creep–recovery cycle includes a 1 s creep procedure and a 9 s recovery procedure. Based on recorded strain, the recovery rate (*R*) at 3.2 kPa and non-recoverable creep compliance (*J_nr_*) at 3.2 kPa of the sample at different stress levels can be yielded via the following equation. Three parallel tests were conducted for each set of samples.

(4) $$R=\frac{\varepsilon_{p}-\varepsilon_{u}}{\varepsilon_{p}}\times 100\%$$

(5) $$J_{nr}=\frac{\varepsilon_{u}}{\sigma }$$

where $\varepsilon_{p}$ is the peak strain at 1 s in each cycle; $\varepsilon_{u}$ is the unrecovered strain at 10 s in each cycle; and σ is the stress level.

## 4. Results and Discussion

### 4.1. Appropriate Short-Term Ageing Scheme for HMB

Essentially, identifying the short-term ageing procedure is a matter of determining which ageing protocol results in the same bitumen ageing degree as the bitumen ageing degree in the short-term ageing bitumen mixture. As such, we attempt to suggest an appropriate short-term ageing solution for modified bitumen through comparing the chemical molecular distribution as well as the rheological properties of bitumen obtained by gradient temperature short-term ageing tests and binders in the short-term-aged mixtures.

#### 4.1.1. Chemical Molecular Distribution of Bitumen and Bitumen in the Mixture

**Figure 4:**
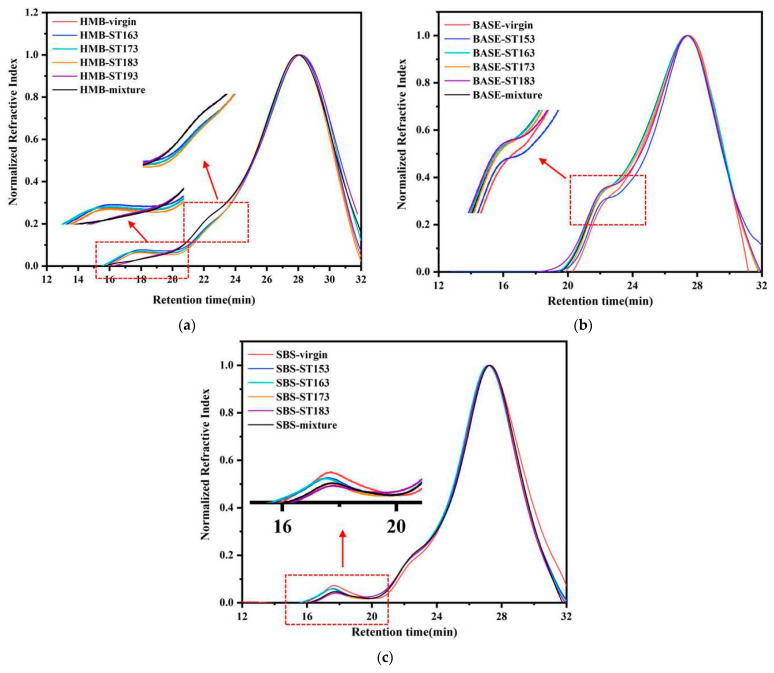
GPC chromatogram of the bitumen and bitumen mixture samples: (**a**) HMB; (**b**) base bitumen; (**c**) SBS-modified bitumen.

Figure 4 illustrates the GPC chromatogram of the bitumen and bitumen mixture samples.

For HMB, the chromatogram showed peaks at 16~19 min and 22~24 min. The former is the peak of the polymer used in the production of HMB, while the latter is the peak of the asphaltene fraction of asphalt. As the short-term ageing temperature of HMB increases from 163 °C to 183 °C, the peaks at 16–19 min and 22–24 min in the GPC chromatograms of short-term-aged HMB show no significant changes. Until the short-term ageing temperature of the HMB increases to 193 °C, the GPC chromatogram of the aged HMB exhibits a gradual decrease in peak heights at 16~19 min, indicating the degradation of the modifiers in HMB. In addition, the GPC chromatogram of the aged HMB exhibits a gradual increase in peak heights at 22~24 min, indicating that the aggregation process occurs in HMB, whereby small molecules tend to agglomerate to form large molecules such as asphaltene. Based on the reported findings, it can be deduced that when the short-term ageing temperature of HMB is lower than 193 °C, the asphalt chemical properties do not change significantly after short-term ageing owing to the poor fluidity of the bitumen. And as can be seen from the figure, the GPC chromatogram of HMB short-term-aged at 193 °C matches well with the bitumen in the mixtures subjected to the STOA test. As such, it can be concluded that 193 °C is a suitable ageing temperature in the TFOT for HMB.

For base bitumen, the chromatogram shows peaks at 22~24 min for the asphaltene phase. As the ageing temperature for base bitumen reaches 163 °C, the peaks of the bitumen chromatogram become high at around 22~24 min. This indicates that molecular conversion occurs in bitumen, where small molecules, such as aromatics, are gradually transformed into larger molecules, such as asphaltene. And the GPC chromatogram of base bitumen short-term-aged at 163 °C is similar to that of the bitumen in short-term-aged mixtures. The above findings suggest that the temperature of TFOT in the existing specification (163 °C) can mimic the ageing phenomenon of base bitumen experienced in the paving process of bitumen pavements.

For SBS-modified bitumen, the chromatogram shows peaks at 16~19 min for the polymer phase and 22~24 min for the asphaltene phase. After ageing, the peak of the SBS bitumen chromatogram gradually decreases at 16~19 min. This can be traced to the degradation of the SBS modifier after ageing. In contrast, the peaks of the aged bitumen chromatograms are elevated at around 22~24 min, suggesting that the aggregation process of small molecules also occurs in SBS-modified bitumen after short-term ageing. It can also be concluded that the increase in ageing temperature mainly affects the degradation of SBS polymers. When the short-term ageing temperatures in the TFOT are set to 153 °C and 163 °C, the SBS modifier peak decreases in the GPC chromatogram, but the decrease is not obvious. When the short-term ageing temperature reaches 173 °C, the SBS modifier peak decreases more noticeably. In addition, the GPC chromatogram of SBS-modified bitumen short-term-aged at 173 °C matches well with the bitumen in short-term-aged mixtures. As such, it can be concluded that 173 °C is a suitable ageing temperature in the TFOT for SBS-modified bitumen. A similar conclusion was reported in the study by Xing et al. [39]. This also illustrates the feasibility of the method used to investigate the appropriate short-term ageing temperature of HMB.

**Figure 5:**
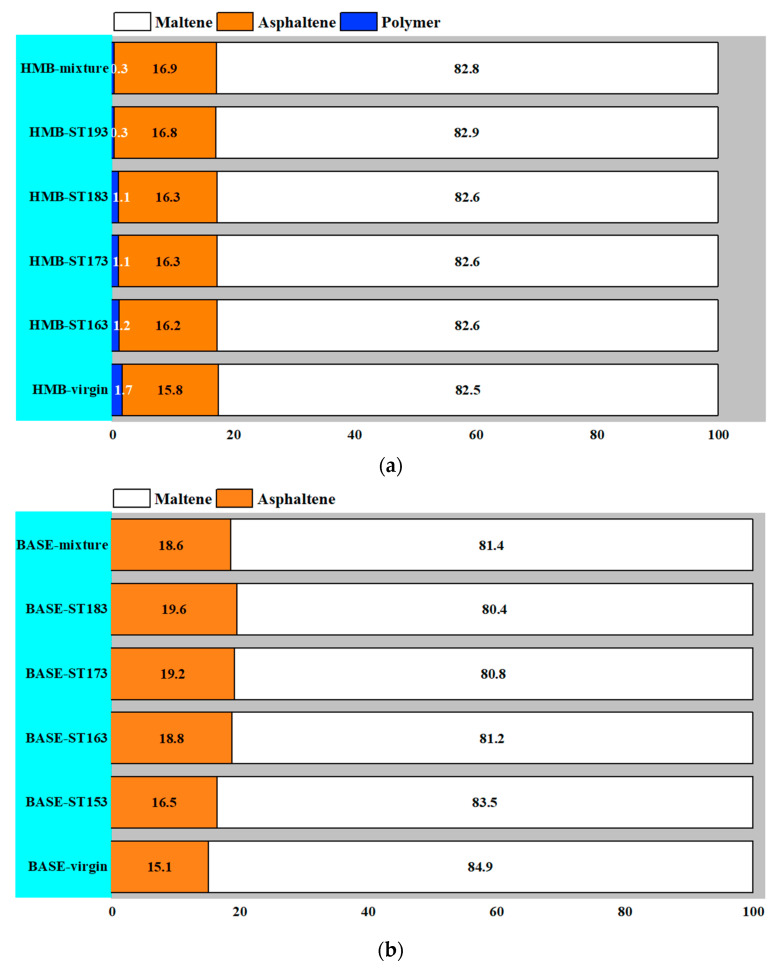

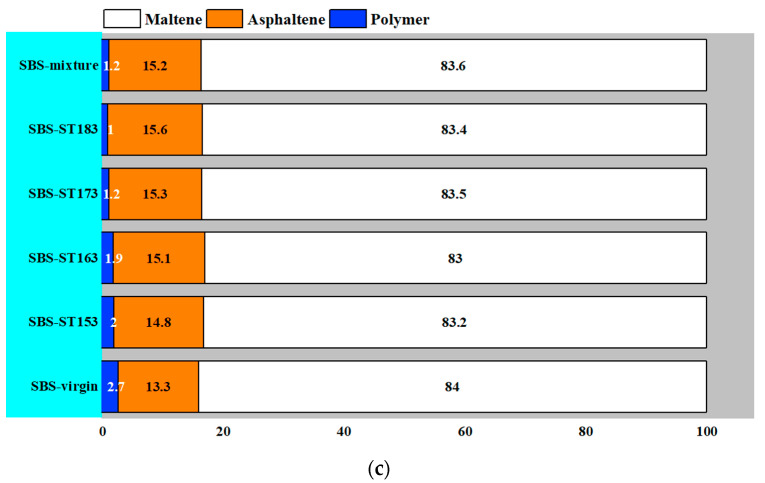
Quantitative results of GPC chromatograms for bitumen samples: (**a**) HMB; (**b**) base bitumen; (**c**) SBS-modified bitumen.

Based on the collected chromatograms, the polymer, asphaltene, and maltene contents of the samples were quantified, and the results are presented in Figure 5.

It can be seen from the figure that the polymer, asphaltene, and maltene contents of HMB in the mixtures after STOA tests match the bitumen after the TFOT at 193 °C. The asphaltene and maltene content of base bitumen in the bitumen mixtures after STOA tests approximately corresponded to base bitumen after the TFOT at 163 °C. And the polymer, asphaltene, and maltene contents of SBS-modified bitumen in the bitumen mixtures after STOA tests generally corresponded to the bitumen after the TFOT at 173 °C. According to the reported results of the GPC tests, it can be summarized that the suitable ageing temperatures for HMB, SBS-modified bitumen, and base bitumen in the TFOT are 193 °C, 173 °C, and 163 °C, respectively.

#### 4.1.2. Rheological Properties of Bitumen and Recovered Bitumen in Bitumen Mixtures

**Figure 6:**
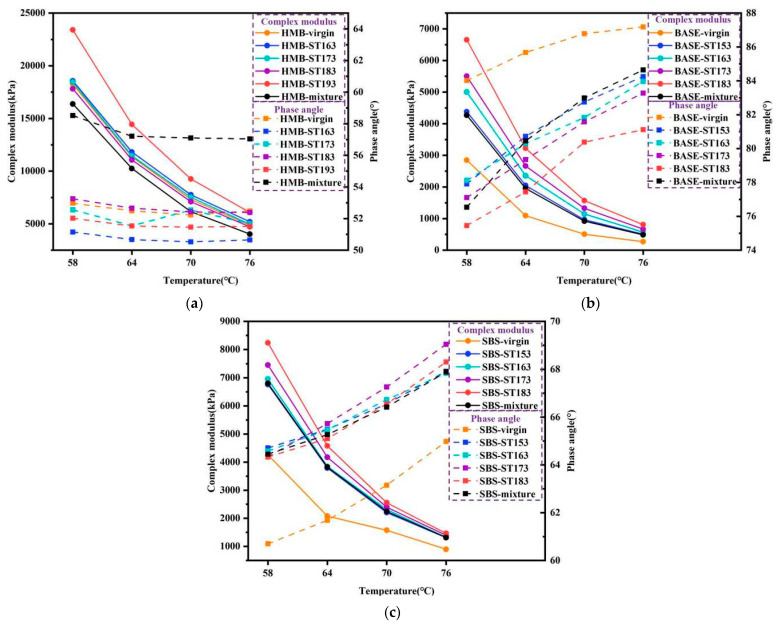
G* and δ of the bitumen samples: (**a**) HMB; (**b**) base bitumen; (**c**) SBS-modified bitumen.

The G* and δ of different bitumen samples obtained in TS are shown in Figure 6. As can be seen from the figure, the G* at four temperatures of all three bitumen samples increases after short-term ageing. The higher the ageing temperatures, the more pronounced the increase in the G*. In addition, the δ of base bitumen gradually decreases as the ageing temperature increases. For SBS-modified bitumen, with ageing temperature increases, the G* of SBS-modified bitumen also rises. In contrast, the increase in the short-term ageing temperature of the HMB caused no regular change in δ of the HMB.

Through comparing the G* and δ of bitumen TFOT-aged under different temperatures and the recovered binders in HMB mixtures subjected to STOA tests, it can be clearly concluded that the G* and δ of recovered base bitumen in the aged mixtures subjected to STOA tests roughly match the base bitumen after the TFOT at 163 °C. The G* and δ of recovered SBS-modified bitumen in the short-term-aged mixtures generally corresponded to the SBS-modified bitumen after the TFOT at 163 °C. Based on the reported findings, it can be proposed that for base bitumen, the TFOT at 163 °C provides a closer simulation of short-term-aged phenomena for the base bitumen pavement. For SBS-modified bitumen, the TFOT at 173 °C provides a superior simulation of the short-term-aged phenomenon for the SBS-modified bitumen pavement. The appropriate short-term ageing temperatures for bitumen yielded through comparing bitumen’s rheological properties are consistent with conclusions yielded in the GPC test. This validates the feasibility of the two approaches to some extent. However, the G* of the recovered HMB in the short-term-aged mixtures is obviously lower than that of the HMB aged by the TFOT. And the δ of the recovered HMB in the short-term-aged mixtures is noticeably higher than that of the HMB aged by the TFOT. This is due to the fact that the HMB used in this study contains PNB, which comprises a certain amount of ash in the bitumen. The presence of the ash leads to a considerable increase in the G* of bitumen. But the extraction and recovery process is unable to recover the ash in the bitumen, which makes the difference between the rheological properties of recovered binders in HMB mixtures following STOA tests and the HMB larger. The above results suggest that it is not feasible to measure the bitumen properties in HMB mixtures using conventional extraction and recovery methods. Meanwhile, GPC technology permits direct testing of HMB in the mixtures without extraction and recovery processes. As such, based on the GPC quantification results, the final short-term ageing temperature in the TFOT for HMB is determined to be 193 °C.

### 4.2. Investigation on the Ageing Behaviour of HMB

After determining the suitable short-term ageing temperatures for base bitumen, SBS-modified bitumen, and HMB, TFOTs were carried out according to the decided ageing temperatures. Following this, additional 20 h, 40 h and 60 h PAV tests were conducted to investigate the long-term ageing behaviour of HMB. Similarly, the long-term ageing behaviour of base bitumen and SBS-modified bitumen was also analyzed for comparison purposes.

#### 4.2.1. GPC Test Results of Bitumen Following Long-Term Ageing

**Figure 7:**
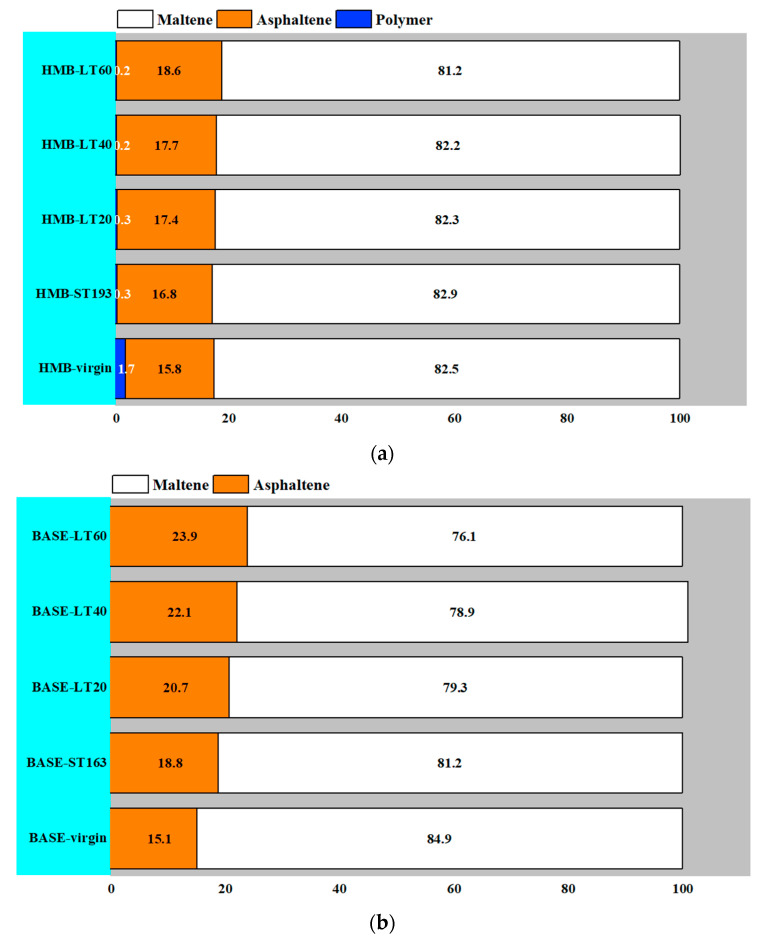

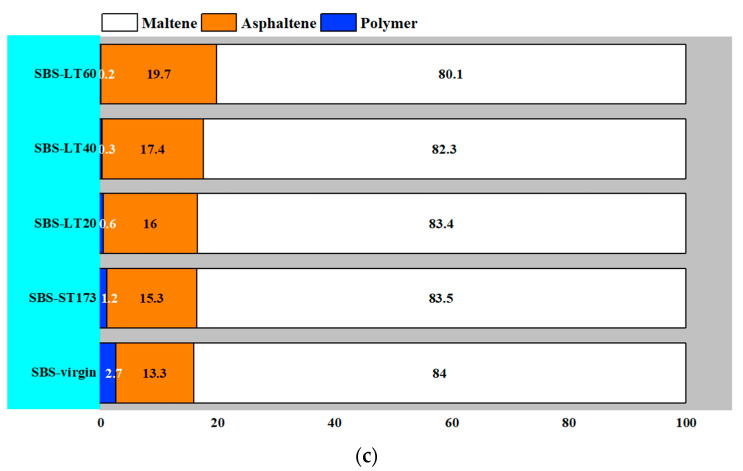
Quantitative results of GPC tests for (**a**) HMB; (**b**) base bitumen; (**c**) SBS-modified bitumen.

The quantitative results of base bitumen, SBS-modified bitumen, and HMB before and after long-term ageing in the GPC test are shown in Figure 7.

It can be seen from the figure that the polymer content of HMB shows no change following long-term ageing, while the asphaltene content of bitumen increases. And the increase in asphaltene content is more pronounced with the increase in the ageing period. This is explained by the polymer in the HMB degrading almost completely after short-term ageing. During the long-term ageing process, the ageing of the bitumen phase dominates. Small molecules such as the aromatic fraction in bitumen continue to polymerise into large molecules, increasing the content of asphaltene in HMB. Similar results are achieved for base bitumen and SBS-modified bitumen, with a significant increase in the asphaltene content of the bitumen after long-term ageing. At the identical long-term ageing time, the increase in asphaltene content in HMB is smaller than that of the base bitumen and SBS-modified bitumen. This may be due to the presence of ash in the HMB mitigating the ageing rate of the bitumen phase in HMB.

#### 4.2.2. TS Test Results of Bitumen Following Long-Term Ageing

**Figure 8:**
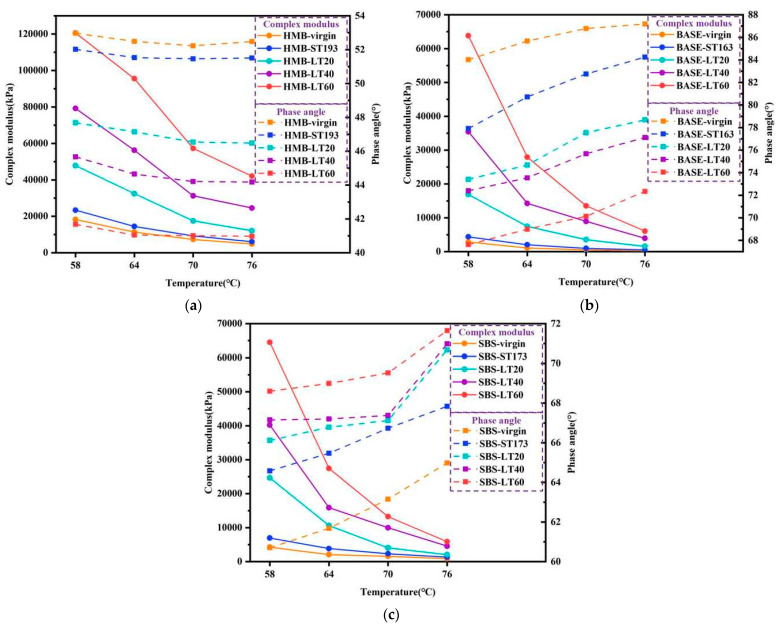
TS test results of bitumen before and after long-term ageing: (**a**) HMB; (**b**) base bitumen; (**c**) SBS-modified bitumen.

The TS test results of bitumen before and after long-term ageing are shown in Figure 8. It can be concluded that for HMB, the G* of HMB at different temperatures increases as the long-term ageing progresses. The explanation for this is that the increase in asphaltene content of long-term-aged HMB strengthens the internal frictional resistance to molecular motion, which in turn leads to an increase in the G* of HMB. The generation of polar oxygen-containing functional groups such as carbonyl and sulfoxide groups after ageing also leads to an increase in the G* of bitumen. In addition, the δ of HMB gradually decreases as the ageing period increases. And the changes in G* and δ are more pronounced after long-term ageing of HMB compared to short-term ageing, suggesting that the effect of long-term ageing on HMB properties is more noticeable. For base bitumen and SBS-modified bitumen, the rheological properties also show regular alterations after long-term ageing. Compared to base bitumen and SBS-modified bitumen, HMB shows less variation in rheological properties after long-term ageing.

#### 4.2.3. MSCR Test Results of Bitumen Following Long-Term Ageing

**Figure 9:**
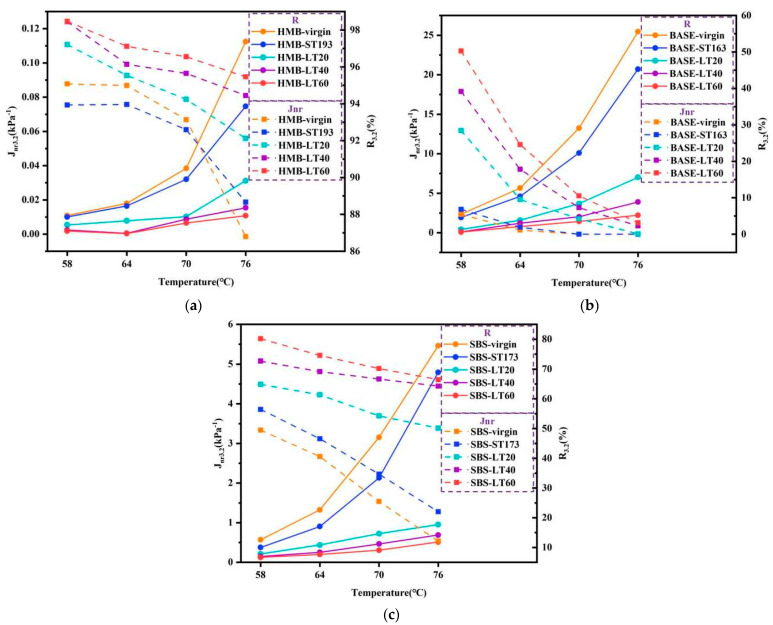
*R*_3.2_ and *J_nr_*_3.2_ of bitumen: (**a**) HMB; (**b**) base bitumen; (**c**) SBS-modified bitumen.

Figure 9 shows the calculated *R*_3.2_ and *J_nr_*_3.2_ of bitumen following long-term ageing in the MSCR test. Non-recoverable creep compliance (*J_nr_*) is used as an assessment indicator of the resistance of bitumen to permanent deformation under repeated loading conditions. It is reported that the *J_nr_*_3.2_ of the bitumen exhibits a good correlation with the rutting depth of bitumen mixtures. For HMB, the *J_nr_*_3.2_ of HMB gradually decreases with the increasing ageing periods, indicating that the resistance of aged HMB to permanent deformation is enhanced. And the effect of long-term ageing is more pronounced for the *J_nr_*_3.2_ of HMB than that of the short-term ageing process. The recovery rate (*R*) reflects the elastic deformation properties of the materials. A better *R* for bitumen samples indicates better elastic deformation properties of the bitumen. As the ageing of HMB progresses, the *R_3.2_* of HMB gradually increases, which also reveals that the ability of the aged bitumen to resist permanent deformation becomes stronger. Similarly, the effect of long-term ageing is more significant for the *R*_3.2_ of HMB than that of the short-term ageing process. In addition, the *J_nr_*_3.2_ and *R*_3.2_ of base bitumen and SBS-modified bitumen show a similar variation pattern due to ageing as that of HMB. At the same ageing conditions, the *J_nr_*_3.2_ of HMB is smaller than that of base bitumen and SBS-modified bitumen, and the *R*_3.2_ is larger than that of base bitumen and SBS-modified bitumen. In addition, it should be noted that the unusually high R values observed for base bitumen at elevated temperatures may be attributed to rotor slippage, and this phenomenon will be thoroughly investigated and validated in subsequent studies. The above results suggest that the high-temperature performance of HMB is better than that of base bitumen and SBS-modified bitumen.

#### 4.2.4. Investigation on the Ageing Resistance of HMB

Referring to the existing studies [50,51], the ageing indexes of base bitumen, SBS-modified bitumen, and HMB were calculated to assess bitumen’s ageing resistance The higher the ageing index of bitumen, the weaker the ageing resistance of the bitumen. The specific formulas are as follows.

(6) $$CAI=\frac{G_{aged}^{*}}{G_{unaged}^{*}}$$

(7) $$AAI=\frac{A_{\mathrm{aged}}}{A_{unaged}}$$

where *CAI* and *AAI* are ageing indexes; $G_{unaged}^{*}$ is the complex modulus of unaged bitumen at 58 °C; $G_{aged}^{*}$ is the complex modulus of aged bitumen at 58 °C; $A_{\mathrm{unaged}}$ is the asphalt content of the unaged bitumen; and $A_{\mathrm{aged}}$ is the asphalt content of the aged bitumen.

Table 3 shows the calculated results of ageing indexes for three kinds of bitumen. It can be concluded that no matter which ageing index is used for evaluation, the ageing indexes of the three types of bitumen in descending order are those for base bitumen, SBS-modified bitumen, and HMB. As such, it can be concluded that the ageing resistance of HMB is better than that of base bitumen and bitumen. This might be attributed to the ash in the HMB retarding the ageing process of the bitumen.

### 4.3. Correlation Between Chemical Size Distribution and Rheological Properties of HMB

To investigate the influence of the chemical molecular weight distribution on the rheological properties, based on the functional expression in Equation (8), correlations linking polymer and asphaltene contents yielded in the GPC test with a 58 °C complex modulus and non-recoverable creep compliance at 3.2 kPa of HMB were constructed.

(8) $$y=A+Bx_{1}+Cx_{2}$$

where *y* represents the rheological properties of HMB; *x*_1_ represents the polymer content of HMB; *x*_2_ represents the asphaltene content of HMB; and *A*, *B* and *C* are fitting parameters.

The polymer content, asphaltene content, G* and *J_nr_*_3.2_ of HMB with different ageing levels were substituted into Equation (8) to obtain the corresponding fitted parameters; the results are shown in Table 4.

As can be seen from the table, the correlations between GPC results and the rheological properties of HMB are good. In comparison, there is a better correlation between the chemical molecular weight distribution and its G*, with the highest R^2^ at 0.956. In addition, the fitting parameter B is lower than C for both G* and *J_nr_*_3.2_, suggesting that asphaltene content has a greater influence on the rheological properties of HMB than polymer content. As mentioned above, it is impractical to measure the rheological properties of the bitumen in HMB mixtures using extraction and recovery methods, since the above process is unable to recover the ash in HMB [44,48]. As an alternative, GPC technology allows us to test the chemical molecular weight distribution of binders in HMB mixtures without the need for extraction processes. In future studies, it will be possible to obtain the rheological properties of bitumen in HMB mixtures by directly testing the chemical molecular weight distribution of the bitumen in the mixtures based on the correlations established in this study. This offers the chance to solve the problem that the traditional method of testing bitumen properties in mixtures by extraction and recovery methods is not applicable to HMB.

## 5. Conclusions

The present study investigated the suitable TFOT ageing temperatures for HMB, base bitumen, and SBS-modified bitumen by comparing the chemical molecular weight distribution and rheological properties of bitumen aged under different temperatures and those of bitumen in the mixtures subjected to STOA tests. We also analyzed the evolution of the chemical molecular weight distribution and rheological properties of bitumen after short-term and long-term ageing processes, and established the correlations between the chemical molecular weight distribution and G* and *J_nr_*_3.2_ of HMB. According to the reported results, the main conclusions are as follows:

1. Due to the inherently high viscosity of HMB, conventional TFOT ageing at lower temperatures does not produce significant ageing effects. This study establishes that raising the TFOT temperature to 193 °C results in an ageing level that closely aligns with that observed in HMB mixtures subjected to STOA tests, thus providing a more representative laboratory ageing protocol for HMB.
2. The presence of mineral ash in PNB-based HMB prevents direct rheological testing of the recovered bitumen using standard extraction and recovery methods, highlighting a key limitation in conventional binder characterization techniques for HMB-containing mixtures.
3. As ageing progresses, polymer degradation and oxidation reactions lead to a decrease in polymer content, phase angle (δ), and non-recoverable creep compliance (*J_nr_*_3.2_) while increasing asphaltene content, complex modulus (G*), and percent recovery (*R*_3.2_). Notably, the impact of long-term ageing is more pronounced than that of short-term ageing, underscoring the need for enhanced ageing resistance strategies in HMB formulations.
4. Compared with base bitumen and SBS-modified bitumen, HMB exhibits superior resistance to ageing and enhanced high-temperature performance, making it a promising material for durable asphalt pavement applications.
5. Correlations between GPC results and the rheological properties of HMB are good. It is possible to obtain the G* and *J_nr_*_3.2_ of bitumen in HMB mixtures by directly testing the chemical molecular weight distribution of the bitumen in HMB mixtures without time-consuming extraction and recovery processes.

It should be noted that only one HMB produced by the incorporation of PNB was used to investigate ageing behaviour in this study. In practice, there are many options for preparing HMB, and future research will investigate the ageing behaviour of HMB using more types of HMB.

## Figures and Tables

**Table 1:** Basic properties of the selected bitumen.

Property	Base Bitumen	SBS-Modified Bitumen	HMB
Penetration (25 °C, 0.1 mm)	68	57	23.7
Softening point (℃)	48.5	85	82.8
Ductility (5 °C, cm)	-	28.5	-
Ductility (15 °C, cm)	>100	-	-
Ductility (25 °C, cm)	-	-	62.5
Ash content	-	-	19.7

**Table 2:** Ageing protocol and identification of bitumen.

Bitumen Type	Short-Term Ageing	Long-Term Ageing	Identification
HMB	-	-	HMB-virgin
	163 °C TFOT	-	HMB-ST163
	173 °C TFOT	-	HMB-ST173
	183 °C TFOT	-	HMB-ST183
	193 °C TFOT	-	HMB-ST193
	TFOT at suitable temperature	20 h PAV	HMB-LT20
	TFOT at suitable temperature	40 h PAV	HMB-LT40
	TFOT at suitable temperature	60 h PAV	HMB-LT60
Base bitumen	-	-	BASE-virgin
	153 °C TFOT	-	BASE-ST153
	163 °C TFOT	-	BASE-ST163
	173 °C TFOT	-	BASE-ST173
	183 °C TFOT	-	BASE-ST183
	TFOT at suitable temperature	20 h PAV	BASE-LT20
	TFOT at suitable temperature	40 h PAV	BASE-LT40
	TFOT at suitable temperature	60 h PAV	BASE-LT60
SBS-modified bitumen	-	-	SBS-virgin
	153 °C TFOT	-	SBS-ST153
	163 °C TFOT	-	SBS-ST163
	173 °C TFOT	-	SBS-ST173
	183 °C TFOT	-	SBS-ST183
	TFOT at suitable temperature	20 h PAV	SBS-LT20
	TFOT at suitable temperature	40 h PAV	SBS-LT40
	TFOT at suitable temperature	60 h PAV	SBS-LT60

**Table 3:** Ageing indexes of different bitumen types.

Bitumen Type	Ageing Periods	*CAI*	*AAI*
HMB	HMB-LT20	2.62	1.10
	HMB-LT40	4.34	1.12
	HMB-LT60	6.60	1.17
Base bitumen	BASE-LT20	5.79	1.37
	BASE-LT40	9.44	1.46
	BASE-LT60	15.15	1.58
SBS-modified bitumen	SBS-LT20	5.93	1.20
	SBS-LT40	12.45	1.30
	SBS-LT60	22.40	1.48

**Table 4:** Fitting parameters of the model.

Rheological Properties	A	B	C	R^2^
Complex modulus (G*) at 58 °C (MPa)	−996.96	41.46	59.76	0.956
*J_nr_*_3.2_ at 58 °C (kPa^−1^)	0.085	−0.003	−0.005	0.872

## Data Availability

The original contributions presented in this study are included in the article. Further inquiries can be directed to the corresponding author.
